# Supplementary material for: CD1d‐mediated activation of group 3 innate lymphoid cells drives IL‐22 production
Source: EMBO Rep. 2016 Oct 31;18(1):39–47. doi: 10.15252/embr.201642412 (PMC5210076; doi:10.15252/embr.201642412)
Supplement: Supplementary file 1 — Expanded View Figures PDF [file EMBR-18-39-s001.pdf]

Expanded View Figures

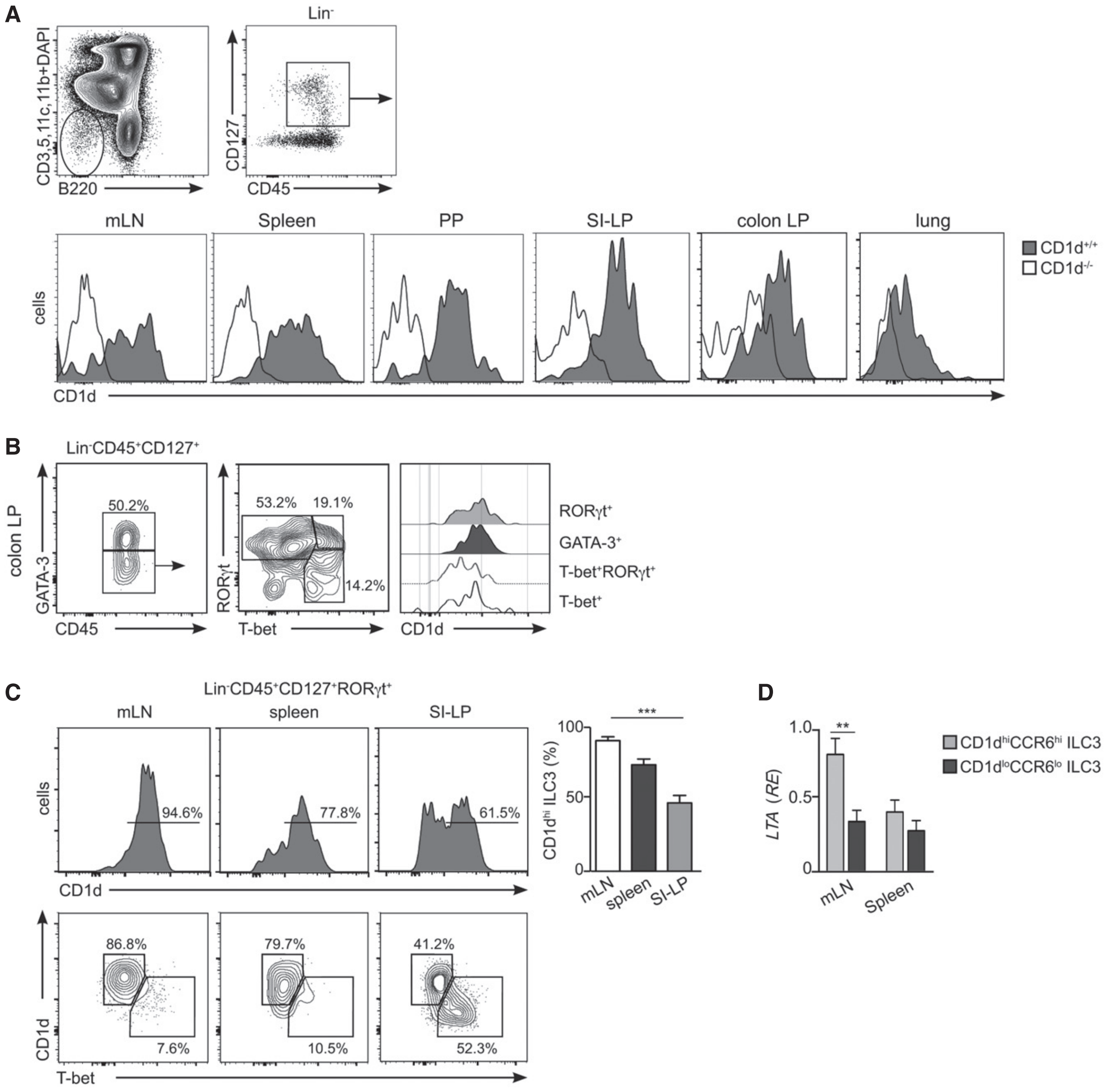

Figure EV1. CD1d expression on ILCs.

A Flow cytometry profiles showing gating strategy and CD1d expression (histogram) in Lin$^-$CD45$^+$CD127$^+$ cells in the depicted tissues from WT (grey filled, n = 4) and CD1d-deficient (empty profile, n = 4) mice.

B Flow cytometry profiles showing gating strategy and CD1d expression (histograms) in T-bet$^+$ (empty profile), T-bet$^+$RORγt$^+$ (dotted line), GATA-3$^+$ (dark grey) and RORγt$^+$ (light grey) ILCs in colonic lamina propria (n = 3). Numbers indicate percentage of cells in the depicted gates.

C Flow cytometry profiles showing expression of CD1d and T-bet in Lin$^-$CD45$^+$CD127$^+$RORγt$^+$ cells from mLN, spleen and SI-LP as indicated (n = 4); numbers indicate percentage of cells. Right panel, percentage of CD1d^{hi} ILC3s in the depicted tissues. ***$P < 0.001$ two-tailed unpaired t-test.

D Relative expression (*RE*) of lymphotoxin-alpha (*LTA*) mRNA (qPCR, normalized to *GAPDH*) in sort-purified CD1d^{hi}CCR6^{hi} (light grey) and CD1d^{low}CCR6^{low} (dark grey) ILC3s isolated from mLN or spleen; **$P < 0.01$, two-tailed unpaired t-test.

Data information: Graphs represent mean ± SEM. Data are from three independent experiments.

 © 2016 The Authors

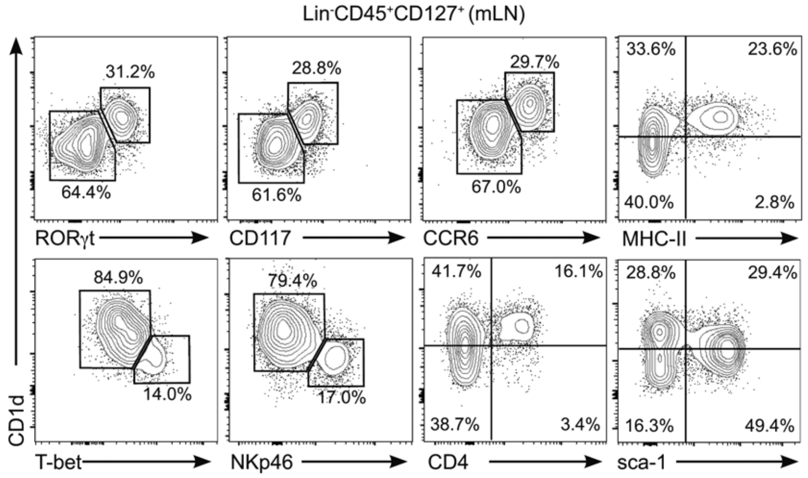

Figure EV2. Phenotype of $CD1d^{+}$ ILCs in mLN.
Flow cytometry profiles showing expression of the depicted markers in $Lin^{-}CD45^{+}CD127^{+}$ cells from mLN. Numbers indicate percentage of cells in the depicted gates. Data represent 2–4 independent experiments.

© 2016 The Authors

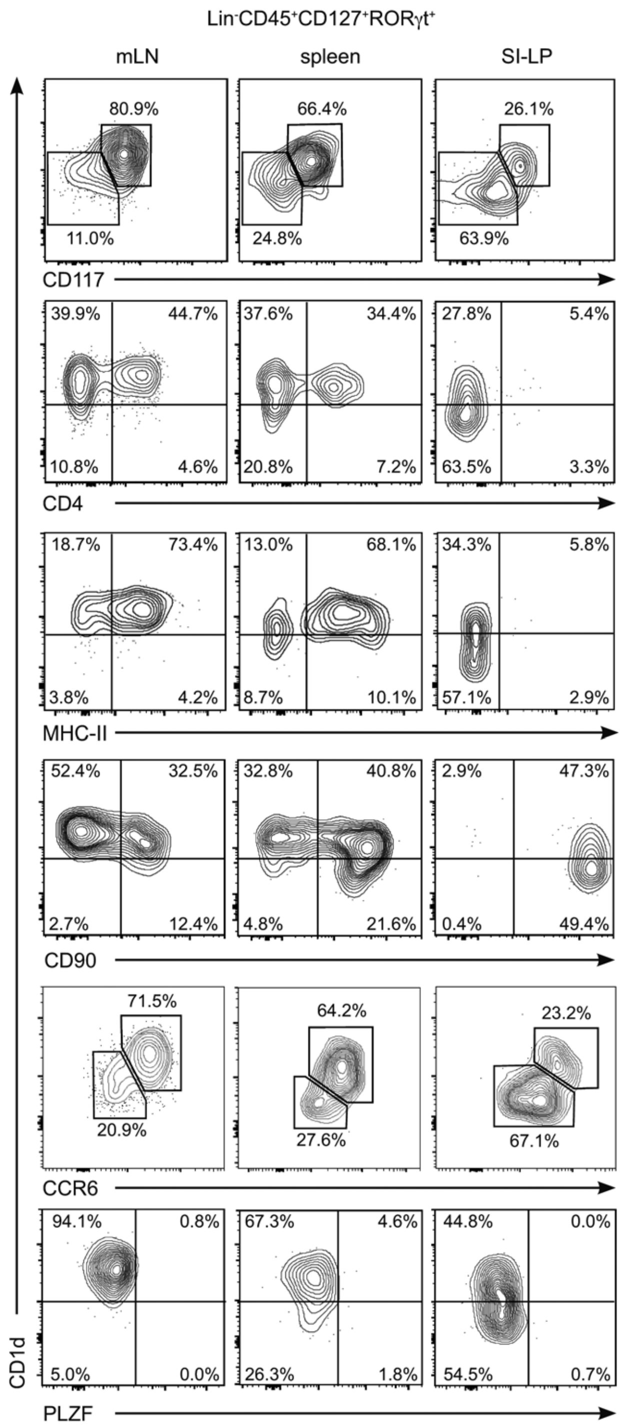

Figure EV3. Phenotype of RORγt$^+$ ILCs.
Flow cytometry profiles showing expression of the depicted markers in RORγt$^+$Lin$^-$CD45$^+$CD127$^+$ ILC3s from mLN, spleen and SI-LP as indicated. Numbers indicate percentage of cells in the depicted gates. Data represent 2–5 independent experiments.

 © 2016 The Authors

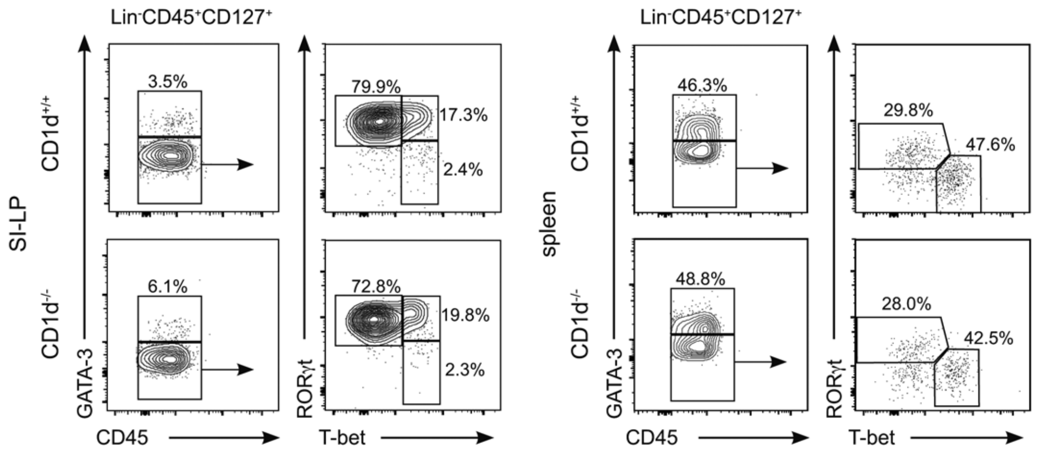

Figure EV4. ILCs in WT and CD1d-deficient mice.
Flow cytometry profiles showing ILC populations from SI-LP and spleen in WT and CD1d-deficient mice as indicated. Data represent four independent experiments.

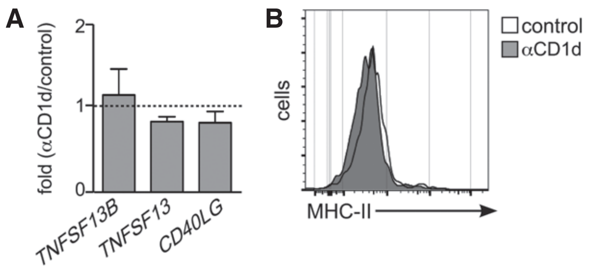

Figure EV5. Effect of CD1d cross-link on ILC3s.

A Fold change in mRNA expression for the indicated cytokines in ILC3s after antibody-mediated CD1d cross-link (n = 4). Gene expression was measured by qPCR and normalized to *GAPDH* and to the mRNA expression levels in control ILC3s. Graphs represent mean ± SEM.

B Flow cytometry profiles showing MHC-II expression on ILC3s in after cross-link with αCD1d antibody (grey) or control (white).

Data information: Data are from four independent experiments.

© 2016 The Authors
